# Systematic review and meta-analysis of the 2010 ASGE non-invasive predictors of choledocholithiasis and comparison to the 2019 ASGE predictors

**DOI:** 10.1007/s12328-021-01575-4

**Published:** 2022-01-24

**Authors:** Louie Wang, Sarah Mirzaie, Tavit Dunnsiri, Formosa Chen, Holly Wilhalme, Ian T. MacQueen, Henry Cryer, Anaar Eastoak-Siletz, Michelle Guan, Callie Cuff, James H. Tabibian

**Affiliations:** 1 David Geffen School of Medicine, University of California, 10833 Le Conte Ave, Los Angeles, CA 90095 USA; 2 Department of Internal Medicine, University of California, Los Angeles, CA USA; 3 Department of Surgery, University of California, Los Angeles, CA USA; 4 Division of Gastroenterology, Department of Surgery, Olive View-UCLA Medical Center, Sylmar, CA USA; 5 Division of Gastroenterology, Department of Medicine, Olive View-UCLA Medical Center, Sylmar, CA USA; 6 Statistics Core, Department of Medicine, University of California, Los Angeles, CA USA

**Keywords:** American Society for Gastrointestinal Endoscopy, Choledocholithiasis, Endoscopic retrograde cholangiopancreatography, ERCP, Cholelithiasis

## Abstract

In 2019, the American Society for Gastrointestinal Endoscopy (ASGE) guideline on the endoscopic management of choledocholithiasis modified the individual predictors of choledocholithiasis proposed in the widely referenced 2010 guideline to improve predictive performance. Nevertheless, the primary literature, especially for the 2019 iteration, is limited. We performed a systematic review with meta-analysis to examine the diagnostic performance of the 2010, and where possible the 2019, predictors. PROSPERO protocol CRD42020194226. A comprehensive literature search from 2001 to 2020 was performed to identify studies on the diagnostic performance of any of the 2010 and 2019 ASGE choledocholithiasis predictors. Identified studies underwent keyword screening, abstract review, and full-text review. The primary outcomes included multivariate odds ratios (ORs) and 95% confidence intervals for each criterion. Secondary outcomes were reported sensitivities, specificities, and positive and negative predictive value. A total of 20 studies met inclusion criteria. Based on reported ORs, of the 2010 guideline “very strong” predictors, ultrasound with stone had the strongest performance. Of the “strong” predictors, CBD > 6 mm demonstrated the strongest performance. “Moderate” predictors had inconsistent and/or weak performance; moreover, all studies reported gallstone pancreatitis as non-predictive of choledocholithiasis. Only one study examined the new predictor (bilirubin > 4 mg/dL and CBD > 6 mm) proposed in the 2019 guideline. Based on this review, aside from CBD stone on ultrasound, there is discordance between the proposed strength of 2010 choledocholithiasis predictors and their published diagnostic performance. The 2019 guideline appears to do away with the weakest 2010 predictors.

## Introduction

Cholelithiasis affects over 25 million individuals in the United States (USA), [1, 2], with associated healthcare expenditures approaching $10 billion annually [3]. Approximately 10–20% of patients with cholelithiasis also have choledocholithiasis (CDL) [4, 5]. Complications of CDL, in turn, include acute pancreatitis (AP) and cholangitis (AC), among others [6]. These conditions increase morbidity and healthcare expenditures, highlighting the importance of early and accurate diagnosis.

Endoscopic retrograde cholangiopancreatography (ERCP) is considered a gold standard diagnostic and a therapeutic modality for choledocholithiasis [7, 8]. However, it carries risk of serious adverse events and should thus be performed selectively, based on pre-test probability [9–11]. In 2010, the American Society for Gastrointestinal Endoscopy (ASGE) guideline on the evaluation of CDL [12] proposed common bile duct (CBD) stone on abdominal ultrasound (US) (US with stone), AC, and total bilirubin (Tbili) > 4 mg/dL as “very strong” predictors;” Tbili 1.8–4 mg/dL and CBD dilation (> 6 mm) as “strong” predictors;” and abnormal liver function test (ALFT), age > 55, and AP as “moderate” predictors.” Likelihood of CDL for any very strong predictor is high, presence of both strong predictor is high, while likelihood of CDL for a single strong predictor or any moderate predictor(s) was not discussed. ERCP was recommend in patients with high likelihood of CDL. Since the publication of this guideline, studies worldwide have examined the performance of these predictors in different patient populations; in some instances, performance has been marginal [13, 14]. In 2019, the ASGE published an updated CDL guideline [15], in which AC, stone on imaging, and the combination of Tbili > 4 mg/dL + CBD dilation were proposed as “high-probability” predictors and ALFT, age > 55, or CBD dilation (without Tbili > 4 mg/dL) as “intermediate.” AP and Tbili 1.8–4 mg/dL were removed from the predictor list, and there were no longer “moderate” strength/probability predictors. In this update, predictors with intermediate probability was recommended additional imaging studies, while predictors with high probability is recommended ERCP, unchanged from the 2010 guideline.

The predictors in the 2019 CDL guideline were modified based on data from five studies examining the performance of the 2010 predictors. For example, the combined predictor “Tbili > 4 mg/dL + CBD > 6 mm” was, for example, recommended given the improved specificity in three studies [13, 16, 17], and AP was removed due to “lack of correlation” [15]. Tbili > 4 mg/dL and 1.8–4 mg/dL were removed for uncertain reasons. Over the past decade, however, there have been additional studies examining the 2010 predictors [13, 16–19], and thus the evidence to support the 2019 modifications may in fact be greater.

The goal of this systematic review is to examine the diagnostic performance of the 2010 ASGE guideline CDL predictors as reported by worldwide studies, and compare the reviewed results with the changes proposed in the newly published 2019 ASGE guideline. This project was not originally designed to involve the need of a meta-analysis. However, an attempt is made to quantify the reporting primary and secondary outcomes in a standardized fashion. We hope that a statistical standardization of primary outcomes will bring further clarity when comparing the predictors’ clinical performances. We believe a more thorough characterization of predictors’ performances in different patient populations can better allow clinicians to apply these guidelines and formulate future modifications and risk-stratification criteria.

## Methods

### Search strategy

Following a methodological process of assessing the literatures, PRISMA 2020 Checklist was used to establish a clear guideline involve both the inclusion and exclusion criteria. The literature review involved a comprehensive search of PubMed, EMBase, SCOPUS, and WEB of SCIENCE for studies published from 2000 to 2020, conducted in adult populations, and written in English (PROSPERO CRD42020194226). In brief, search terms included: choledocholithiasis, cholelithiasis, endoscopic retrograde cholangiopancreatography, ERCP, ASGE, endoscopic US or endosonography, biochemical parameter, CBD, and statistics. Search results were extracted and organized using Zotero 5.0 for further keyword screening after review of duplicates. Two independent reviewers (LW & SM) screened the title and abstracts of the studies identified in the preliminary search were screened, and studies that assessed the diagnostic performance of any ASGE choledocholithiasis predictor was reviewed in full. individually reviewed the abstracts of the screened-in studies to assess the relevance to the study objective. Studies that led to disagreement or uncertainty between the two reviewers were adjudicated by a third reviewer (JT). Afterward, full-text of the included studies was obtained. Full-text reviews were then conducted based on selection criteria alongside data extraction.

The exact search for PubMed database was conducted using: *("Cholelithiasis"[Mesh] OR "Choledocholithiasis"[Mesh] OR "Gallbladder Diseases"[Mesh] OR "Cholecystitis"[Mesh] OR "Cholecystolithiasis"[Mesh]) AND (ercp[MeSH Terms] OR (intraoperative_cholangiogram) OR Endosonography[MeSH Terms] OR Cholangiopancreatography, Magnetic Resonance[Mesh]) AND ((Risk calculator) OR algorithm[MeSH Terms] OR Predictive Value of Tests[MeSH Terms] OR (Predictive) OR (Prognostic Value) OR Quality of Health Care[MeSH Terms]) Filters: Humans, English, Adult: 19* + *years, from 2000 to 2020.*

The exact search for EMBASE database was conducted using: *('Cholelithiasis' OR 'common bile duct stone' OR 'gallbladder disease' OR 'cholecystitis' OR 'cholelithiasis') AND ('endoscopic retrograde cholangiopancreatography' OR 'cholangiography' OR 'endoscopic ultrasonography' OR 'magnetic resonance cholangiopancreatography') AND ('health care quality' OR 'prognostic value' OR (predictive) OR 'predictive value' OR 'algorithm' OR 'risk calculator') AND [adult]/lim AND [humans]/lim AND [2000–2020] /py.*

The exact search for WEB of SCIENCE database was conducted using:

*TS* = *(Cholelithiasis OR Choledocholithiasis OR Gallbladder Diseases OR Cholecystitis OR Cholecystolithiasis) AND (ERCP OR intraoperative_cholangiogram OR Endoscopic ultrasound OR MRCP) AND (Risk calculator OR algorithm OR Predictive OR Prognostic Value) AND LANGUAGE: (English) Refined by: [excluding] RESEARCH AREAS: (PEDIATRICS).*

### Selection criteria

We included original studies that reported statistical findings regarding the predictive value of ASGE-listed predictors in patients with or suspected CDL. Exclusion criteria were as follows:

- Studies that did not include ‘predict’ and ‘associat in the title or abstract
- Studies published due to abstract-only nature, or full-text link not found on databases.
- Studies lacking statistical values for the ASGE predictors and/or focusing on a different outcome, such as the long-term impact of endoscopic CDL management.
- Studies focused on a specific demographic population (pregnancy, elderly, or immunocompromised, etc.)

### Data extraction

The data extraction proceeded with standardized guidelines, focusing on reported statistical quantification of predictor: ORs, univariate vs. multivariate OR analyses, upper and lower 95% confidence interval (CI), *p* values, sensitivity, specificity, PPV, and NPV. The sample size associated with each reported predictor was also extracted.

For ALFT, only studies that literally stated or reported “abnormal liver function tests per ASGE” (or similar) were extracted. Data related to the predictor “US with stone” were extracted if the reporting studies specified procedure as transabdominal US. Tbili levels were reported in a standardized unit of mg/dL. The sample size mean age for the CBD > 6 mm predictor was extracted concerning confounding as CBD dilate associates with aging. Non ASGE predictors were excluded in the extraction. Only the initial set of clinical data, such as lab values, were extracted in cases of multiple sets of data being reported in a study.

### Data analysis

Formulation of conversation are drafted according to the Cochran Handbook [20]. For each predictor in either multivariate or univariate analysis, forest plots were generated from Prism—GraphPad^®^ with a calculated inverse-variance weight (1/Variance). Variance was calculated using the following formula:

$$\frac{1}{{{\text{Weight}}}} = { }\left( {\frac{{\left( {{\text{ln}}\left( {{\text{upper }}95{\text{\% CI}}} \right) - {\text{ln}}\left( {{\text{lower }}95{\text{\% CI}}} \right)} \right)}}{{2{*}1.96}}} \right)^{2} \times {\text{n}}$$

where *n* is the specific sample size reported a study for the specific predictor.

A weighted average OR (WaOR) was then calculated using the reported ORs of the predictors and their calculated inverse-variance weight by the following formula:

$${\text{Wa OR}} = { }\frac{{{\text{sum of }}\left( {{\text{OR }} \times {\text{weight}}} \right)}}{{\text{sum of weight}}}$$

Although our review has grouped the predictor AP and AC on forest plots, studies had an inconstant definition for AP and AC, which are labeled in results. The exact definition of AP and AC from studies are reported in the results.

When examining the predictive value of a criteria, we sought to report OR as the primary outcome. OR exceeding 1 indicates high strength of association between the predictor and CDL. Although OR is not meant for establishing a causation relationship, its strength of association fits our clinical definition of high predictive value in clinical management. Traditionally, ORs are also the calculation standard in statistical analysis. Thus, our review defined a predictor with a high WaOR to have high predictive strength.

Sensitivity, specificity, PPV, and NPV were also extracted as secondary outcomes. If a predictor had high specificity, it then demonstrated a high strength in ruling out the true negatives, thus helping in ruling in the true positives. Similarly, if a predictor has a high PPV, it then demonstrates a strong strength in ruling in the true positives from the total positives. Statistically, if a predictor has a strong predictive value, it shall be reported with high specificity and PPV.

## Results

### Extraction results

**Fig. 1:**
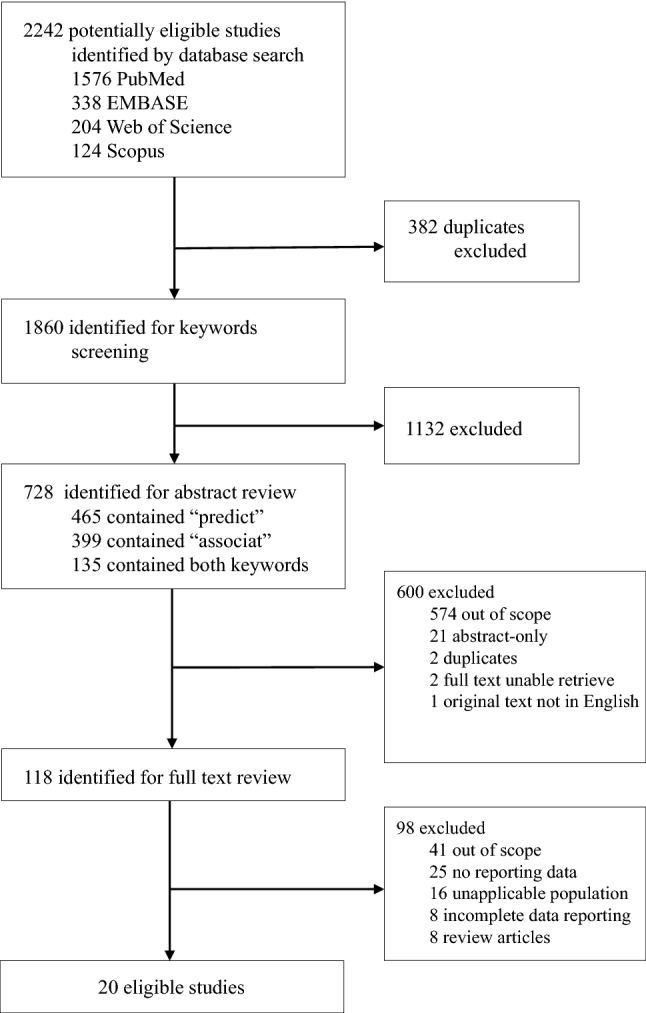
A flowchart of the literature search, review, and selection process of this review

A total of 2242 studies were initially extracted. After excluding 382 duplicates, 1860 studies were identified for keyword screening. After excluding 1132 studies not containing “predict” or “associate” in the abstract, 728 studies were included for detailed abstract review conducted by two reviewers (LW and SM), with a third reviewer (JT) arbitrating. This process eliminated 600 studies, yielding 118 studies for full-text review. After full-text review, a total of 98 studies focusing on endpoints that were unrelated to the predictive value of ASGE predictors (41), did not report statistical data (25), had a population not primarily related to CDL (16), did not have complete data reported for extraction (8), or were reviews in nature(8) were excluded. In total 20 studies were included (Fig. 1). Aiming to improve internal validity, we followed the PRISMA 2020 check-list to exclude all studies that met the exclusion criteria. The final 20 studies all met with the ordinally designed methodological quality. They had a median sample size of 165, ranging between 18 and 2055. Three studies were published between 2000 and 2010, two between 2010 and 2015, and 15 after 2015. Among the primary outcomes, Suarez et al. is the only study reported ORs in multivariable instead of multivariate analysis. Regarding study types, 11 studies were reported as a retrospective and 9 as a prospective cohort. Eight studies were conducted in the United States (USA), two in South Korea and India each, and the studies remaining studies were individually from China, Lebanon, Lithuania, Portugal, Saudi Arabia, Spain, Sweden, and Turkey.

### Statistical findings

#### Overview of extraction results

**Fig. 2:**
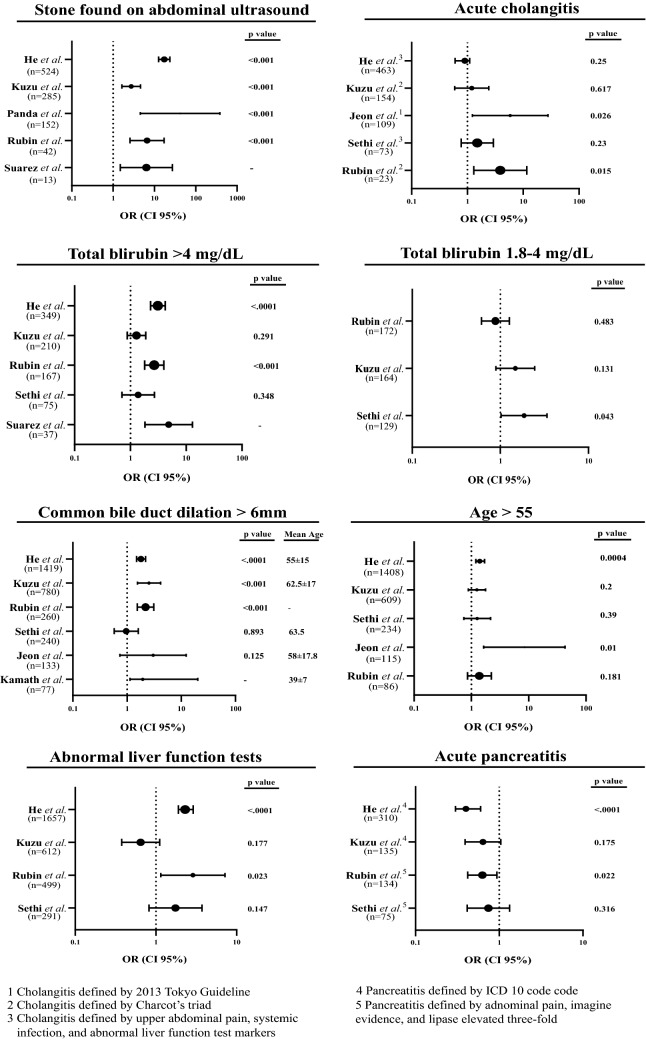
Forest plots of studies with multivariate analyzed OR reporting

**Fig. 3:**
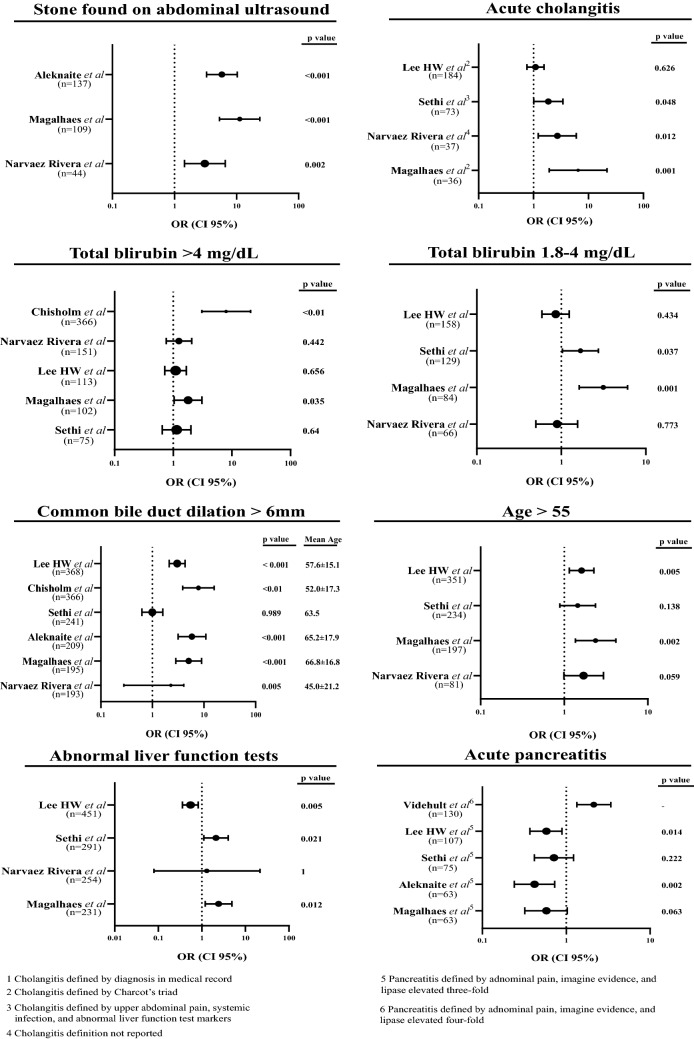
Forest plots of studies with univariate analyzed OR reporting

Of the 20 included studies that reported predictive statistical data for CDL, 13 reported ORs and their associated statistical data (95% CI and *p* values) for at least one ASGE CDL predictor. In these studies, six reported multivariate-analyzed ORs and six univariate-analyzed ORs. One study reported both multivariate- and univariate-analyzed ORs. A forest plot was generated for each ASGE predictor with studies that reported ORs using multivariate (Fig. 1) and univariate analysis (Fig. 2), and WaOR for each predictor in both multivariate and univariate analyses were calculated (Table 1). Detailed statistical findings regarding the OR-reported studies are presented in Figs. 2 and 3. Statistical details for Figs. 2 and 3 are reported in Tables 2 and 3, respectfully. All 20 studies reported sensitivity, specificity, PPV, and NPV for at least one ASGE predictor, as shown in Table 4. A meta-analysis for data reported in Table 4 was not attempted given concern for a lack of statistical significance data without reported range of confidence intervals, difficulty in attributing weights, and reporting bias. Detailed information regarding each included study were composed on Table 5.

**Table 1:** Weighted average odds ratio for univariate and multivariate analyzed studies

2010 ASGE definition	Predictors	Univariate analysis average weighted OR (WaOR)	Multivariate analysis average weighted OR (WaOR)
Very strong predictor	US with stone	5.566	8.618
	Acute Cholangitis	2.500	2.290
	Tbli > 4 mg/dL	1.417	2.619
Strong predictor	Tbli 1.8–4 mg/dL	1.458	1.278
	CBD > 6 mm	3.697	1.900
Moderate predictor	ALFT	1.540	1.896
	Age > 55	1.742	1.567
	Acute Pancreatitis	0.811	0.620

Sample sizes reported were *relevant* sample sizes, as not all studies had samples comprised entirely of (suspected) CDL patients

**Table 2:** Detailed information of multivariate analyzed studies shown in Fig. 2

Predictor	Study	*n*	OR	CI lower 95%	CI upper 95%	*p* value	Variance	Weight (%)
US with stone	He et al. [21]	524	17.3	12.6	23.8	< .0001	13.8	7.25
	Kuzu et al. [22]	285	2.74	1.63	4.60	< 0.001	19.9	5.01
	Panda et al. [23]	152	42	4.56	386	0.001	195	0.51
	Rubin et al. [19]	43	6.65	2.57	17.2	< 0.001	10.1	9.90
	Suarez et al. [24]	13	6.4	1.5	27.3	–	7.12	14.04
Acute cholangitis	He et al. [21]	463	0.9	0.6	1.1	0.25	11.1	9.03
	Kuzu et al. [22]	154	1.2	0.59	2.42	0.617	19.8	5.04
	Jeon et al. [25]	109	5.84	1.23	27.8	0.026	68.9	1.45
	Sethi et al. [26]	73	1.50	0.77	2.93	0.23	8.46	11.82
	Rubin et al. [19]	23	3.88	1.3	11.6	0.015	7.16	13.97
Tbli > 4 mg/dL	He et al. [21]	349	3.1	2.3	4.2	< .0001	8.24	12.14
	Kuzu et al. [22]	210	1.29	0.88	1.89	0.291	7.99	12.52
	Rubin et al. [19]	167	2.67	1.8	3.97	< 0.001	6.80	14.71
	Sethi et al. [26]	75	1.38	0.70	2.704	0.348	8.84	11.31
	Suarez et al. [24]	37	4.85	1.82	12.9	–	9.25	10.81
Tbli 1.8–4 mg/dL	Rubin et al. [19]	172	0.88	0.61	1.27	0.483	6.02	16.61
	Kuzu et al. [22]	164	1.48	0.89	2.45	0.131	10.9	9.13
	Sethi et al. [26]	129	1.86	1.02	3.38	0.043	12.1	8.28
CBD > 6 mm	He et al. [21]	1419	1.80	1.50	2.20	< .0001	13.5	7.38
	Kuzu et al. [22]	780	2.54	1.55	4.16	< 0.001	49.5	2.02
	Rubin et al. [19]	260	2.19	1.54	3.12	< 0.001	8.4	11.86
	Sethi et al. [26]	241	0.97	0.58	1.61	0.89	16.3	6.14
	Jeon et al. [25]	133	3.02	0.74	12.3	0.13	68.5	1.46
	Kamath et al. [27]	77	1.95	1.13	20.2	–	41.6	2.41
ALFT	He et al. [21]	1657	2.30	1.90	2.90	< .0001	19.3	5.19
	Kuzu et al. [22]	612	0.65	0.38	1.12	0.177	47.1	2.12
	Rubin et al. [19]	499	2.88	1.15	7.18	0.023	108.9	0.92
	Sethi et al. [26]	291	1.75	0.82	3.72	0.147	43.2	2.32
Age > 55	He et al. [21]	1408	1.40	1.20	1.70	0.0004	11.1	9.00
	Kuzu et al. [22]	609	1.25	0.89	1.76	0.20	18.6	5.38
	Sethi et al. [26]	234	1.26	0.74	2.16	0.39	17.4	5.76
	Jeon et al. [25]	115	8.46	1.65	43.3	0.01	79.9	1.25
	Rubin et al. [19]	86	1.38	0.86	2.23	0.181	5.08	19.7
Acute pancreatitis	He et al. [21]	310	0.40	0.30	0.60	< .0001	9.69	10.3
	Kuzu et al. [22]	135	0.64	0.39	1.05	0.175	8.46	11.8
	Rubin et al. [19]	134	0.63	0.42	0.94	0.022	5.66	17.7
	Sethi et al. [26]	75	0.74	0.41	1.33	0.316	6.64	15.1

**Table 3:** Detailed information of univariate analyzed studies shown in Fig. 3

Predictor	Study	*n*	OR	CI lower 95%	CI upper 95%	*p* value	Variance	Weight (%)
US with stone	Aleknaite et al. [28]	137	5.81	3.3	10.3	< 0.001	11.5	8.72
	Magalhaes et al. [18]	109	11.3	5.32	23.8	< 0.001	15.9	6.28
	Nárvaez Rivera et al. [14]	44	3.09	1.45	6.58	0.002	6.55	15.27
Acute cholangitis	Lee et al. [29]	184	1.09	0.76	1.55	0.626	6.08	16.44
	Magalhaes et al. [18]	36	6.48	1.93	21.8	0.001	13.8	7.26
	Nárvaez Rivera et al. [14]	37	2.71	1.22	6.01	0.012	6.12	16.33
	Sethi et al. [26]	73	1.85	1.01	3.412	0.048	7.07	14.14
Tbli > 4 mg/dL	Chisholm et al. [30]	366	7.97	3.06	20.8	< 0.01	87.5	1.14
	Nárvaez Rivera et al. [14]	151	1.25	0.76	2.07	0.442	9.87	10.14
	Lee et al. [29]	113	1.1	0.72	1.67	0.656	5.21	19.21
	Magalhaes et al. [18]	102	1.79	1.04	3.08	0.035	7.85	12.78
	Sethi et al. [26]	75	1.14	0.65	2.00	0.64	6.13	16.31
Tbli 1.8–4 mg/dL	Lee et al. [29]	158	0.86	0.59	1.24	0.434	5.67	17.63
	Sethi et al. [26]	129	1.69	1.03	2.75	0.037	8.07	12.39
	Magalhaes et al. [18]	84	3.15	1.63	6.08	0.001	9.47	10.56
	Nárvaez Rivera et al. [14]	66	0.89	0.5	1.56	0.773	5.56	17.98
CBD > 6 mm	Lee et al. [29]	368	3.03	2.12	4.30	< 0.001	12.0	8.35
	Chisholm et al. [30]	366	7.83	3.87	15.8	< 0.01	47.3	2.11
	Sethi et al. [26]	241	1.00	0.63	1.59	0.99	13.5	7.41
	Aleknaite et al. [28]	209	5.85	3.15	10.9	< 0.001	21.0	4.77
	Magalhaes et al. [18]	195	5.06	2.85	8.99	< 0.001	16.7	5.97
	Nárvaez Rivera et al. [14]	193	2.28	0.28	4.08	0.01	90.1	1.11
ALFT	Lee et al. [29]	451	0.55	0.36	0.83	0.005	20.5	4.88
	Sethi et al. [26]	291	2.12	1.12	4.01	0.021	30.8	3.36
	Nárvaez Rivera et al. [14]	254	1.30	0.08	21.60	1	518.1	0.19
	Magalhaes et al. [18]	231	2.43	1.20	4.90	0.01	29.8	3.25
Age > 55	Lee et al. [29]	351	1.61	1.15	2.26	0.005	10.4	9.59
	Sethi et al. [26]	234	1.45	0.89	2.37	0.138	14.7	6.81
	Magalhaes et al. [18]	197	2.37	1.36	4.15	0.002	16.0	6.27
	Nárvaez Rivera et al. [14]	81	1.70	0.99	2.94	0.059	6.25	16.0
Acute pancreatitis	Videhult et al. [17]	130	2.13	1.34	3.41	–	7.38	13.6
	Lee et al. [29]	107	0.58	0.37	0.89	0.014	5.36	18.6
	Sethi et al. [26]	75	0.72	0.42	1.23	0.222	5.64	17.7
	Aleknaite et al. [28]	63	0.42	0.24	0.73	0.002	5.07	19.7
	Magalhaes et al. [18]	63	0.58	0.32	1.03	0.063	5.60	17.9

**Table 4:** Studies reported sensitivity, specificity, PPV, and NPV for each ASGE predictor

Predictor	Study	*n*	Sensitivity (%)	Specificity (%)	Positive predictive value (%)	Negative predictive value (%)
US with stone	Yang et al. [31]	926	35.7	97.9	58.1	94.9
	He et al. [21]	524	44	97	91	73
	Kuzu et al. [22]	285	36.6	85.3	90.5	26
	Jagtap et al. [16]	174	62	99.6	98.3	87.9
	Aleknaite et al. [28]	137	51.3	84.6	86.2	48.3
	Magalhaes et al. [18]	109	55.9	89.9	91.7	50.3
	Bose et al. [32]	88	50	97	87	82
	Adams et al. [13]	65	21.8	93.5	70.8	62.3
	Nárvaez Rivera et al. [14]	44	23	91	77	48
	Rubin et al. [19]	43	13	98	88	47
	Suarez et al. [24]	13	14.1	97.1	76.9	61.9
Acute cholangitis	He et al. [21]	463	20	84	44	61
	Videhult et al. [17]	323	22	70	9	88
	Lee et al. [29]	184	32.4	69.6	59.8	42.4
	Kuzu et al. [22]	154	18.9	88.6	86.3	22.1
	Bose et al. [32]	88	42	100	100	80
	Jagtap et al. [16]	71	22.8	99	88.7	78.1
	Nárvaez Rivera et al. [14]	37	19	92	76	47
	Magalhaes et al. [18]	36	18.4	96.6	91.7	37
	Rubin et al. [19]	23	7	98	83	45
Tbli > 4 mg/dL	He et al. [21]	349	22	94	69	65
	Kuzu et al. [22]	210	25.2	82.6	84.7	22.4
	Rubin et al. [19]	167	41	79	72	51
	Nárvaez Rivera et al. [14]	151	61	44	59	47
	Lee et al. [29]	113	20	81.5	60.2	42.1
	Magalhaes et al. [18]	102	42.5	70.8	74.5	37.8
	Suarez et al. [24]	37	29.6	84.3	56.8	63.2
	Chan et al. [33]	19	39	95	93	44
Tbli 1.8–4 mg/dL	Rubin et al. [19]	172	32	63	54	43
	Kuzu et al. [22]	164	19.5	85.3	83.5	21.6
	Lee et al. [29]	158	25.9	71.2	55.7	40.71
	Magalhaes et al. [18]	84	61.1	66.6	75	51.2
	Nárvaez Rivera et al. [14]	66	25	73	55	53
CBD > 6 mm	He et al. [21]	1419	75	63	57	79
	Kuzu et al. [22]	780	70.9	23.9	82	70.4
	Jagtap et al. [16]	434	69.9	68.5	44.5	86.4
	Lee et al. [29]	368	73.8	51.9	68.2	58.6
	Rubin et al. [19]	260	58	61	66	53
	Aleknaite et al. [28]	209	92.5	32.2	71.3	70.2
	Magalhaes et al. [18]	195	83.8	49.4	76.9	60.3
	Nárvaez Rivera et al. [14]	193	82	33	62	59
ALFT	He et al. [21]	1657	77	50	50	77
	Kuzu et al. [22]	612	71.1	39.6	81.8	26.4
	Rubin et al. [19]	499	98	7	57	68
	Lee et al. [29]	451	73.2	16.9	55.2	31.1
	Nárvaez Rivera et al. [14]	254	99	1	57	50
	Magalhaes et al. [18]	231	89.9	21.3	69.7	51.3
	Jagtap et al. [16]	205	90.2	38.5	34.6	91.6
Age > 55	He et al. [21]	1408	60	54	46	67
	Kuzu et al. [22]	609	69.6	35.3	80.4	23.3
	Lee et al. [29]	351	65	46.5	62.3	48.7
	Jagtap et al. [16]	205	37	69.5	30.4	75.4
	Magalhaes et al. [18]	197	79.3	38.2	72.1	47.9
	Rubin et al. [19]	86	18	86	63	45
	Nárvaez Rivera et al. [14]	81	37	85	65	47
Acute pancreatitis	Jagtap et al. [16]	408	23.2	55.9	15.7	66.6
	He et al. [21]	310	10	85	29	59
	Kuzu et al. [22]	135	13.6	78.8	71.1	19.3
	Rubin et al. [19]	134	22	69	48	41
	Videhult et al. [17]	130	21	90	20	90
	Lee et al. [29]	107	15	77	47.7	39.3
	Bose et al. [32]	88	12	96	60	72
	Nárvaez Rivera et al. [14]	80	20	54	36	34
	Magalhaes et al. [18]	63	20.1	69.7	57.1	30.2
	Adams et al. [13]	57	41.7	68.9	43.9	67
	Suarez et al. [24]	56	55.6	76.3	52.6	78.4
Tbli > 4 mg/dL + CBD > 6 mm	He et al. [21]	267	19	96	78	58

Sample sizes reported were *relevant* sample sizes, as not all studies had samples comprised entirely of (suspected) CDL patients

**Table 5:** Characteristics of included studies

Study	Publication year	Journal	Study type	Total sample size	Study demographic	Gold standard
Adams et al. [13]	2015	Gastrointestinal Endoscopy	Retrospective cohort	498	USA	ERCP
Aleknaite et al. [28]	2018	United European Gastroenterology Journal	Retrospective cohort	350	Lithuania	ERCP & IOC
Bose et al. [32]	2001	Surgery Today	Prospective cohort	88	India	ERCP & IOC
Chan et al. [33]	2008	The American Journal of Surgery	Retrospective cohort	182	USA	ERCP
Chisholm et al. [30]	2019	Gastrointestinal Endoscopy	Prospective cohort	366	USA	ERCP
He et al. [21]	2017	Gastrointestinal Endoscopy	Prospective cohort	2724	China	ERCP
Jagtap et al. [16]	2020	Endoscopy	Prospective cohort	1042	India	ERCP
Jeon et al. [25]	2017	Gut and Liver	Retrospective cohort	200	Korea	ERCP
Kamath et al. [27]	2016	Indian Journal of Gastroenterology	Prospective cohort	275	Saudi Arabia	ERCP
Kuzu et al. [22]	2017	HPB	Retrospective cohort	1074	Turkey	ERCP
Lee et al. [29]	2019	Hepatobiliary & Pancreatic Diseases International	Retrospective cohort	754	Korea	ERCP
Magalhaes et al. [18]	2015	World Journal of Gastrointestinal Endoscopy	Retrospective cohort	268	Portugal	ERCP
Nárvaez Rivera et al. [14]	2016	Spanish Journal of Gastroenterology	Prospective cohort	256	Spain	ERCP
Panda et al. [23]	2018	World Journal of Surgery	Retrospective cohort	152	USA	ERCP
Rahal et al. [34]	2017	European Journal of Gastroenterology & Hepatology	Retrospective cohort	354	Lebanon	ERCP & IOC
Rubin et al. [19]	2013	Digestive Endoscopy	Retrospective cohort	1080	USA	ERCP
Sethi et al. [26]	2016	Digestive Endoscopy	Prospective cohort	336	USA	ERCP
Suarez et al. [24]	2016	Surgical Endoscopy	Prospective cohort	173	USA	ERCP
Videhult et al. [17]	2011	HPB	Prospective cohort	1171	Sweden	IOC
Yang et al. [31]	2008	Surgical Endoscopy	Retrospective cohort	2225	USA	Cholecystectomy

Sample sizes reported were *total* sample sizes, including patients not entirely of (suspected) CDL patients. Specific *relevant* sample size is reported in Table 2, 3, 4 for each specific predictor

#### Stone found on abdominal ultrasound

Five studies reported multivariate analyzed ORs with a WaOR of 8.62. Sample sizes ranged from 13 [24] to 524 [21]. He et al. [21] (OR 17.3; [95% CI 12.6–23.8]) reported the highest OR, while Kuzu et al*.* [22] (OR 2.74; [95% CI 1.63–4.60]) reported the lowest OR. Rubin et al*.* [19] (OR 6.65; [95% CI 2.58–17.2]) carried the most inverse variance weight (9.9%).

Three studies reported univariate analyzed ORs with a weighted average of 5.57. Sample sizes ranged from 44 [14] to137 [28]. Magalhaes et al*.* [18] (OR, 11.3; [95% CI 5.32–23.8]) reported the highest OR, while Nárvaez Rivera et al*.* [14] (OR, 3.09; [95% CI 1.45–6.58]) reported the lowest OR and carried the most inverse-variance weight (15%) among these three studies.

Eleven studies reported sensitivity (mean 37%, range 13–62%), specificity (mean 94%, range 85–100%), PPV (mean 81%, range 58–98%), and NPV (mean 62%, range 26–95%) for this predictor.

#### Acute cholangitis

Five studies reported multivariate analyzed ORs with a WaOR of 2.29. Sample sizes ranged from 23 [19] to 463 [21]. Jeon et al*.* [25] (OR 5.84; [95% CI 1.23–27.8]) reported the highest OR, while He et al*.* [21] (OR 0.9; [95% CI 0.6–1.1]) reported the lowest OR. Rubin et al*.* [19] (OR 3.88; [95% CI 1.3–11.6]) carried the most inverse-variance weight (14%).

Five studies reported univariate analyzed ORs with a WaOR of 2.5. Sample sizes ranged from 73 [26] to 184 [29]. Magalhaes et al*.* [18] (OR 6.48; [95% CI 1.93–21.8]) reported highest OR, while He et al*.* [21] (OR 1.09; [95% CI 0.76–1.55]) reported the lowest OR. Lee et al*.* [29] (OR 1.09; [95% CI 0.76–1.55]) carried the most inverse-variance weight (16%) among these three studies.

Nine studies reported sensitivity (mean 23%, range 18–32%), specificity (mean 89%, range 70–100%), PPV (mean 71%, range 9–100%), and NPV (mean 56%, range 22–88%) for this predictor.

#### Total bilirubin

##### *Tbili* > *4 mg/dL*

Five studies reported multivariate analyzed ORs with a WaOR of 2.62. Sample sizes ranged from 37 [24] to 349 [21]. Suarez et al*.* [24] (OR 4.85; [95% CI 1.82–12.9]) reported the highest OR, while Kuzu et al*.* [22] (OR 1.29; [95% CI 0.88–1.89]) reported the lowest OR. Rubin et al*.* [19] (OR 2.67; [95% CI 1.8–3.97] carried the most inverse-variance weight (15%).

Five studies reported univariate analyzed ORs with a WaOR of 1.42. Sample sizes ranged from 75 [26] to 366 [30]. Chisholm et al*.* [30] (OR 7.97; [95% CI 3.06–20.8]) reported the highest OR, while Lee et al*.* [29] (OR 1.1; [95% CI 0.72–1.67]) reported the lowest OR and carried the most inverse-variance weight (19%) among these three studies.

Eight studies reported sensitivity (mean 35%, range 20–61%), specificity (mean 79%, range 44–95%), PPV (mean 71%, range 57–93%), and NPV (mean 47%, range 22–65%) for this predictor.

##### Tbili 1.8–4 mg/dL

Three studies reported multivariate analyzed ORs with a WaOR of 1.28. Sample sizes ranged from 129 [26] to 172 [19]. Sethi et al*.* [26] (OR, 1.86; [95% CI, 1.02–3.38]) reported the highest OR, while Rubin et al*.* [19] (OR, 0.88; [95% CI, 0.61–1.27]) reported the lowest OR and carried the most inverse-variance weight (17%).

Four studies reported univariate analyzed ORs with a WaOR of 1.46. Sample sizes ranged from 66 [14] to 158 [29]. Magalhaes et al*.* [18] (OR 3.15; [95% CI 1.63–6.08]) reported the highest OR, while Lee et al*.* [29] (OR 0.86; [95% CI 0.59–1.24]) reported the lowest OR. Nárvaez Rivera et al*.* [14] (OR 0.89; [95% CI 0.5–1.56]) carried the most inverse-variance weight (18%) among these three studies.

Five studies reported sensitivity (mean 33%, range 20–61%), specificity (mean 72%, range 63–73%), PPV (mean 65%, range 54–84%), and NPV (mean 42%, range 43–53%) for this predictor.

#### CBD > 6 mm

Six studies reported multivariate analyzed ORs with a WaOR of 1.9. Sample sizes ranged from 77 [27] to 1419 [21]. Jeon et al*.* [25] (OR 3.02; [95% CI 0.74–12.3]) reported the highest OR, while Sethi et al*.* [26] (OR 0.97; [95% CI 0.58–1.61]) reported the lowest OR. Rubin et al*.* [19] (OR 2.19; [95% CI 1.54- 3.12]) carried the most inverse-variance weight (12%).

Six studies reported univariate analyzed ORs with a WaOR of 3.70. Sample sizes ranged from 193 [14] to 368[29]. Chisholm et al*.* [30] (OR 7.83; [95% CI 3.87–15.8]) reported highest OR, while Sethi et al*.* [26] (OR 1; [95% CI 0.63–1.59]) reported the lowest OR. Lee et al*.* [29] (OR 3.03; [95% CI 2.12–4.3]) carried the most inverse-variance weight (8.4%) among these three studies.

Eight studies reported sensitivity (mean 76%, range 58–93%), specificity (mean 48%, range 24–69%), PPV (mean 66%, range 45–82%), and NPV (mean 67%, range 53–86%) for this predictor.

#### Abnormal liver function test (ALFT)

Four studies reported multivariate analyzed ORs with a WaOR of 1.90. Sample sizes ranged from 291 [26] to 1657 [21]. Rubin et al*.* [19] (OR 2.88; [95% CI 1.15–7.18]) reported the highest OR, while Kuzu et al*.* [22] (OR 0.65; [95% CI 0.38–1.12]) reported the lowest OR. He et al*.* [21] (OR 2.3; [95% CI 1.9–2.9]) carried the most inverse-variance weight (5.2%).

Four studies reported univariate analyzed ORs with a WaOR of 1.54. Sample sizes ranged from 231[18] to 451 [29]. Magalhaes et al*.* [18] (OR 2.43; [95% CI 1.2–4.9]) reported the highest OR, while Lee et al*.* [29] (OR 0.55; [95% CI 0.36–0.83]) reported the lowest OR and carried the most inverse-variance weight (4.9%) among these three studies.

Seven studies reported sensitivity (mean 85%, range 71–99%), specificity (mean 25%, range 1–59%), PPV (mean 58%, range 35–82%), and NPV (mean 56%, range 26–92%) for this predictor.

#### Age > 55

Five studies reported multivariate analyzed ORs with a WaOR of 1.57. Sample sizes ranged from 86 [19] to 1408 [21]. Jeon et al*.* [25] (OR 8.46; [95% CI 1.65- 43.3]) reported the highest OR, while Kuzu et al*.* [22] (OR 1.25; [95% CI 0.89–1.76]) reported the lowest OR. Rubin et al*.* [19] (OR 1.38; [95% CI 0.86–2.23]) carried the most inverse variance weight (20%).

Four studies reported univariate analyzed ORs with a WaOR of 1.74. Sample sizes ranged from 81 [14] to 351 [29]. Magalhaes et al*.* [18] (OR 2.37; [95% CI 1.36–4.15]) reported highest OR, while Sethi et al. (OR 1.45; [95% CI 0.89–2.37]) reported the lowest OR. Nárvaez Rivera et al*.* [14] (OR 1.7; [95% CI 0.99–2.94]) carried the most inverse-variance weight (16%) among these three studies.

Seven studies reported sensitivity (mean 53%, range 18–79%), specificity (mean 59%, range 35–86%), PPV (mean 60%, range 30–80%), and NPV (mean 51%, range 23–75%) for this predictor.

#### Acute pancreatitis

Four studies reported multivariate analyzed ORs with a WaOR of 0.62. Sample sizes ranged from 75 [26] to 310 [21]. Sethi et al*.* [26] (OR 0.74; [95% CI 0.41–1.33]) reported the highest OR, while He et al*.* [21] (OR 0.4; [95% CI 0.3–0.6]) reported the lowest OR. Rubin et al*.* [19] (OR 0.63; [95% CI 0.42–0.94]) carried the most inverse variance weight (18%).

Five studies reported univariate analyzed ORs with a WaOR of 0.811. Sample sizes ranged from 63 [18, 28] to 130 [17]. Videhult et al*.* [17] (OR 2.13; [95% CI 1.34–3.41]) reported the highest OR, while Aleknaite et al*.* [28] (OR 0.42; [95% CI 0.24–0.73]) reported the lowest OR. Lee et al*.* [29] (OR 0.58; [95% CI 0.37–0.89]) carried the most inverse-variance weight (19%) among these three studies.

Eleven studies reported sensitivity (mean 23%, range 10–56%), specificity (mean 75%, range 54–96%), PPV (mean 44%, range 16–71%), and NPV (mean 54%, range 19–90%) for this predictor.

#### Total bilirubin > 4 mg/dL and CBD > 6 mm

He et al*.* [21] was the only study that reported diagnostic performance of this newly proposed predictor from the 2019 ASGE guideline. The study reported a sample size of 267, sensitivity 19%, specificity 96%, PPV 78%, and NPV 58%.

## Discussion

In this systematic review, we aimed to identify studies that examined the predictive performance of the 2010 ASGE guideline CDL predictors and helped contributing to the changes proposed on the 2019 ASGE guideline. As the result, we excluded all publications that did not report ‘predict’ and ‘associate’ in their title or abstract. Our key findings include: (i) “US with stone” demonstrated outstanding predictive value compared to other predictors, (ii) CBD > 6 and AC demonstrated similar predictive value for CDL, potentially calling into question the modification made in the 2019 ASGE guideline, (iii) Tbili is not reliable for CDL when used alone, and (iv) AP demonstrated a negative predictive trend for CDL.

A high-level summary of the findings of this study is provided on Table 6. With a high WaOR in both univariate and multivariate analyses (Table 1), our review showed that “US with stone” had a significantly higher predictive value for CDL than all other predictors, including their peer predictors Tbili > 4 and AC. With a low reported sensitivity, this predictor is less helpful in ruling out CDL; however, a high reported specificity provides strength for ruling in CDL. The forest plots (Figs. 1, 2) and *p* values further support this observation. “US with stone” has also been reported to have high specificity (94%) and PPV (81%), significant for ruling in CDL. Spontaneous passage of stone before ERCP may explain why PPV, though high, is typically not 100%. Although AC and Tbili > 4 mg/dL did not perform as strongly as “US with stone,” they demonstrated a relatively higher predictive value than all other predictors. However, Tbili > 4 mg/dL had a lower predictive value, demonstrated by its WaOR in univariate analysis, when used alone. This finding is not unexpected, as multiple factors and pre-existing conditions can lead to elevated Tbili levels.

**Table 6:** Comparison of various CDL predictors between the 2010 and the 2019 ASGE guidelines with study resulted recommendation and comments

Predictors	2010 ASGE guideline CDL predictor category	2019 ASGE guideline CDL predictor category	Comment based on study result
US with stone	Very strong	High	Highest WaOR of all predictors
Acute cholangitis	Very strong	High	Lower WaOR than US with stone; however, still relatively higher WaOR than other predictors
Tbili > 4 mg/dL	Very strong	Not reported	Equivalent multivariate analysis WaOR as compared to acute cholangitis
Tbili > 4 mg/dL + CBD > 6 mm	Not reported	High	Insufficient data to comment; suspect higher specificity than either alone
Tbili 1.8–4 mg/dL	Strong	Not reported	Relatively low WaOR, equivalent to moderate predictors reported in 2010 ASGE guideline. However, limited studies
CBD > 6 mm	Strong	Intermediate	High WaOR on univariate analysis, low WaOR on multivariate analysis. Relatively high sensitivity and NPV
ALFT	Moderate	Intermediate	Low WaOR, nonspecific
Age > 55 years	Moderate	Intermediate	Low WaOR, nonspecific
Acute pancreatitis	Moderate	Not reported	WaOR < 1, indicates a negative predictor of CDL

It is presumed that the term “likelihood” reported in the 2010 ASGE guideline and “probability” reported in the 2019 ASGE guideline have analogous meanings and, similarly, that the strength qualifiers (e.g. “very strong”) reported in the 2010 ASGE guideline and probability (e.g. “high”) qualifiers reported in the 2019 ASGE guideline have analogous meanings

Despite being in the same group of “strong predictor,” CBD > 6 mm demonstrated a stronger predictive value than Tbili 1.8–4 mg/dL (Table 1). It’s worth noting that CBD > 6 mm might be more useful in a younger patient population, as CBD diameter can gradually increase with age. We also found that CBD > 6 mm had high sensitivity (76%) and similar PPV (66%) and NPV (67%). This finding potentially indicates that the absence of CBD dilation is helpful in ruling out CDL. Based on the WaOR, Tbili 1.8–4 mg/dL demonstrated less predictive value than its peer and performed worse than the “moderate predictors,” ALFT and age > 55. This finding potentially invalidates the role of Tbili 1.8–4 as a strong predictor; however, our review found fewer studies that reported data for Tbili 1.8–4 mg/dL, which limits interpretation. Moreover, relatively high specificity (72%) was observed with Tbili 1.8–4 mg/dL, potentially indicating some predictive value.

ALFT is loosely defined. It can refer to any abnormal liver biochemical value other than bilirubin. Our review demonstrated that ALFT has a similar predictive value as CBD > 6 mm in multivariate analysis and has relatively higher sensitivity, potentially indicating some added clinical value. However, when used alone, the predictive value was relatively low. Overall, the predictive value of ALFT is difficult to determine given its imprecise definition. Additional studies are needed to investigate the role of different biochemical markers and their temporal trend in CDL prediction.

Age > 55 demonstrated minimal predictive value with its relatively weak WaOR, similar to ALFT. Furthermore, Table 4 shows no standout with any of the four other statistical factors, further limiting its predictive value. With regard to AP, our review revealed a WaOR < 1, indicating a negative association, suggested that (persistence of) CDL is perhaps less likely in patients with AP. Concordantly, the 2019 ASGE guideline no longer includes AP as a CDL predictor or risk criterion.

In summary, the 2019 ASGE guideline designates AC and “US with stone” as a high-risk predictors and no longer has Tbili > 4 mg/dL alone as a “strong” predictor, instead requiring it to be in combination with CBD dilation to be considered a composite “high-risk” predictor. The 2019 guideline also downgrades CBD dilation alone to the “intermediate-risk” group and removes AP and Tbili 1.8–4 mg/dL altogether. Our study lacked predictive data for the grouped predictor “CBD dilation with Tbili > 4 mg/dL” except for one study [21]. The recommendation in the presence of a “high-risk” predictor is to proceed to ERCP, while additional imaging modalities are recommended for “intermediate-risk” predictors. Overall, our review supports the conclusions drawn by many of the subject studies that the 2010 ASGE guideline CDL predictors had gaps in accuracy and evidence base.

This systematic review has limitations. Common with systematic reviews, missing data continues to be a challenge with the extraction process. To make efforts in retrieve any missing data and avoid attrition bias, the finalized data extraction results were crossed checked by reviewers (SM and LW). Despite including studies from a variety of geographic and demographic populations, some populations were better represented than others. Our study attempted to perform meta-analysis on a heterogeneous population, given the variability in clinical courses and data reporting. This heterogeneity creates subjective bias potentially confounding the scandalization process. Additionally, we excluded all non-English studies. These factors may decrease the generalizability of our findings. Additionally, most of the studies reported were from the last decade and the USA, which may further limit generalizability. Some predictors, for example Tbili 1.8–4 mg/dL, had a relative paucity of data to analyze, thus limiting interpretation. Furthermore, as the review heavily involved literature search and data extraction, this process could generate human error. In the risk of bias assessment, we recognize that our study is subject to selective outcome reporting, as our exclusion criteria might omit to report related to our scope of the investigation, attrition bias, as limited by human error in the data extraction process. Lastly, our study is prone to detection biases and publication error associated with forest plots, as the weight of each standardized OR is dependent to its relative sample size, which is subject to further confounding and biases within the reporting studies. Our secondary outcomes, including sensitivity, specificity, PPV, and NPV, lacked a standardized statistical analysis due to limitations of the available data in the included studies.

Our study reviewed the predictive value of individual non-invasive CDL predictors. However, in clinical practice, CDL diagnosis is often made with a combination of factors. A liver function panel reports multiple predictors, including Tbili and liver enzymes. A right-upper-quadrant abdominal ultrasound not only assesses for stone presence but also attempts to visualize the degree of CBD dilation. As our findings indicated, some predictors excel in ruling out CDL with high sensitivity and NPV, while others excel in ruling in CDL with high specificity and PPV. Thus, it is important clinicians rely on more than one predictor in managing patients with suspicion of CDL. Though further study remains needed and dynamic changes in predictors should be considered on a case-by-case clinical basis, until such data become available, our review largely supports the modifications made to CDL predictors in the 2019 ASGE guideline as evidence-based.
